# In Vitro Antileishmanial and Antischistosomal Activities of Anemonin Isolated from the Fresh Leaves of *Ranunculus multifidus* Forsk

**DOI:** 10.3390/molecules26247473

**Published:** 2021-12-10

**Authors:** Betelhem Sirak, Kaleab Asres, Asrat Hailu, Mthandazo Dube, Norbert Arnold, Cecile Häberli, Jennifer Keiser, Peter Imming

**Affiliations:** 1 Department of Pharmaceutical Chemistry and Pharmacognosy, School of Pharmacy, College of Health Sciences, Addis Ababa University, Addis Ababa P.O. Box 1176, Ethiopia; betelhem.sirak@amu.edu.et; 2 Department of Pharmacy, College of Medicine and Health Sciences, Arba Minch University, Arba Minch P.O. Box 21, Ethiopia; 3 Department of Microbiology, Immunology and Parasitology, Faculty of Medicine, College of Health Sciences, Addis Ababa University, Addis Ababa P.O. Box 1176, Ethiopia; asrat.hailu@aau.edu.et; 4 Department of Bioorganic Chemistry, Leibniz Institute of Plant Biochemistry, Weinberg 3, D-06120 Halle (Saale), Germany; mdube@ipb-halle.de (M.D.); Norbert.Arnold@ipb-halle.de (N.A.); 5 Swiss Tropical and Public Health Institute, Socinstr. 57, CH-4051 Basel, Switzerland; cecile.haeberli@swisstph.ch (C.H.); jennifer.keiser@swisstph.ch (J.K.); 6 University of Basel, CH-4051 Basel, Switzerland; 7 Institut fuer Pharmazie, Martin-Luther-Universitaet Halle-Wittenberg, Kurt-Mothes-Str. 3, 06120 Halle (Saale), Germany

**Keywords:** *Ranunculus multifidus*, fresh leaves, antileishmanial activity, *Leishmania aethiopica*, *Leishmania donovani*, promastigotes, amastigotes, macrophage, selectivity index, anemonin

## Abstract

Leishmaniasis and schistosomiasis are neglected tropical diseases (NTDs) infecting the world’s poorest populations. Effectiveness of the current antileishmanial and antischistosomal therapies are significantly declining, which calls for an urgent need of new effective and safe drugs. In Ethiopia fresh leaves of *Ranunculus multifidus* Forsk. are traditionally used for the treatment of various ailments including leishmaniasis and eradication of intestinal worms. In the current study, anemonin isolated from the fresh leaves of *R. multifidus* was assessed for its in vitro antileishmanial and antischistosomal activities. Anemonin was isolated from the hydro-distilled extract of the leaves of *R. multifidus*. Antileishmanial activity was assessed on clinical isolates of the promastigote and amastigote forms of *Leishmania aethiopica* and *L. donovani* clinical isolates. Resazurin reduction assay was used to determine antipromastigote activity, while macrophages were employed for antiamastigote and cytotoxicity assays. Antischistosomal assays were performed against adult *Schistosoma mansoni* and newly transformed schistosomules (NTS). Anemonin displayed significant antileishmanial activity with IC_50_ values of 1.33 nM and 1.58 nM against promastigotes and 1.24 nM and 1.91 nM against amastigotes of *L. aethiopica* and *L. donovani*, respectively. It also showed moderate activity against adult *S. mansoni* and NTS (49% activity against adult *S. mansoni* at 10 µM and 41% activity against NTS at 1 µM). The results obtained in this investigation indicate that anemonin has the potential to be used as a template for designing novel antileishmanial and antischistosomal pharmacophores.

## 1. Introduction

Leishmaniasis is a neglected tropical disease (NTD) infecting the world’s poorest populations. According to WHO [1], an estimated 700,000 to 1,000,000 new cases of leishmaniases occur annually. The disease is endemic and highly prevalent in Ethiopia where people in the highlands are at higher risk of contracting cutaneous leishmaniasis, while visceral leishmaniasis is prevalent mostly in lowland arid areas of the country [2,3,4,5,6]. Efficacy, toxicity, cost and availability of drug are among factors determining the choice of drug to treat leishmaniasis [7]. The effectiveness of currently available drugs is significantly declining due to increased drug resistance, emerging cross resistance, requirements for parenteral administration and/or length of treatment, lack of new drugs with novel mechanisms of action and unavailability of effective vaccine [8,9,10,11,12]. This has highlighted the urgent need to explore traditionally used medicinal plants as a source of new antileishmanial drugs in order to minimize the debilitating impact of the disease.

In Ethiopia, *Ranunculus multifidus* Forsk. (Ranunculaceae) is commonly known by its vernacular names such as ‘*Etse siol*’ (Geez), ‘*Gundi*’ (Amharic), ‘*Tuche* or *Aysmamata*’ (Gamo), ‘*Abba warqe*’ (Afaan Oromo) and ‘*Hogioo*’ (Kaffa) [13,14,15]. In traditional medicine, dried and crushed roots and leaves of *R. multifidus* are used to clear out intestinal worms [16]. The leaves are also applied topically to treat leishmaniasis in different parts of Ethiopia. In some parts of the country the dried leaves are pounded to powder and mixed with honey and applied on wounds caused by visceral leishmaniasis [17], while in other parts fresh leaves are crushed and rubbed on the affected part [18]. However, despite the popularity of the plant in traditional medicine for the control of leishmaniasis and eradication of intestinal worms, no phytochemical or biological studies concerning the activity of the plants against leishmaniasis could be found in the literature. The genus *Ranunculus* has however been established as an anti-inflammatory, analgesic, antiviral, antibacterial, antiparasitic and antifungal agent due to the presence of anemonin [19].

Schistosomiasis is the most widely distributed chronic NTD affecting people living in communities where poor environmental sanitation and inadequate clean water supply are prevalent [20]. Globally, 700 million people are at risk with 240 million in 76 countries infected [21]. Human schistosomiasis is second only to malaria in mortality with an annual death of 200,000 people [22]. In Ethiopia, 5 million people are infected with schistosomiasis and 37.5 million are at risk of infection [23].

The purpose of this study is, therefore, to investigate the in vitro antileishmanial activity of fresh leaves of the plant against the promastigote and intracellular amastigote forms of *L. aethiopica* and *L. donovani* clinical isolates. The report further details antileishmanial and antischistosomal activity of the major compound isolated from the hydro-distilled extract of the fresh leaves of *R. multifidus* characterised as anemonin.

## 2. Results and Discussion

In the current study, fresh leaves of *R. multifidus* were used to prepare extracts in order to mimic the form in which the plant is used in traditional medicine. As the bioactive compounds present in the plant may vary in their polarity, two extraction methods were employed. Cold maceration with 80% methanol was used to extract heat-labile polar and moderately polar compounds, while hydro-distillation was employed to extract thermally stable and relatively non-polar components. Maceration with hydro-alcohol gave 6.4% (*w*/*w*) of brown powder, while hydro-distillation yielded 0.56% (*w*/*w*) yellow coloured irritating oil.

Antileishmanial assay was done on *L. aethiopica* and *L. donovani*, which are the major causes of cutaneous leishmaniasis and visceral leishmaniasis in Ethiopia, respectively [3,6,24]. AlamarBlue^®^ or resazurin (7-hydroxy-10-oxidophenoxazin-10-ium-3-one) reduction assay was used to determine the antileishmanial activity and cytotoxicity of the test substances as the technique permits a simple, rapid, reliable, sensitive, cost-effective method for continuous monitoring of cell cultures [25].

In both antileishmanial and cytotoxicity assays, a positive linear correlation was established between viability of cell and concentrations of test substances (R^2^ = 0.981–0.992). As shown in Table 1, both the hydro-alcoholic and hydro-distilled extracts displayed antileishmanial activity on both the promastigote and amastigote forms of *L. aethiopica* and *L. donovani* with IC_50_ values ranging from 0.49 to 22.12 μg/mL. However, the hydro-distilled extract was found to be significantly (*p* < 0.001) more potent than the hydro-alcoholic extract. Thus, further phytochemical analysis of the former using preparative thin layer chromatography (PTLC) resulted in the isolation of the α,β-unsaturated dilactone anemonin by the method described earlier [26]. The current study disclosed that anemonin possesses strong and comparable growth inhibitory effect against the promastigote and amastigote forms of *L. aethiopica* and *L. donovani* with antipromastigote IC_50_ values of 0.257 μg/mL (1.33 nM) and 0.303 μg/mL (1.58 nM), respectively. Anemonin also inhibited growth of the amastigote form of the parasites at nanomolar concentrations which were much lower than those of the extracts. However, amphotericin B was superior in its potency (*p* < 0.001) to that of anemonin against both the promastigote and amastigote forms of the tested parasites. 

Even though promastigotes and amastigotes evidently vary in their bioenergetics, morphology, gene expression, protein phosphorylation and expression of membrane proteins which result in difference in their susceptibility to test substances [27,28,29], the test substances displayed similar activities against the promastigote and amastigote forms of the tested parasites.

In vitro macrophage cytotoxicity tests revealed that the hydro-alcoholic extract is relatively nontoxic compared to the hydro-distilled extract, anemonin or amphotericin B (Table 2). Haemolysis test also confirmed that the hydroalcoholic extract has a very high LC_50_ value (>1000 µg/mL) indicating its relative safety to red blood cells. Both the hydro-distilled extract and amphotericin B were found to be toxic to macrophages and caused red blood cells lysis at much lower concentrations. Overall, the hydro-alcoholic extract appeared to be less toxic than the hydro-distilled extract. This is congruent with previous studies that reported the aqueous and dichloromethane: methanol (1:1) extracts of *R. multifidus* do not have cytotoxic effect on methyl thiazolyl tetrazolium (MTT) cellular viability assay against the human embryonic kidney epithelial cell line (104% cell growth at 100 μg/mL) [30].

The in vitro macrophage cytotoxicity assay indicated that anemonin and amphotericin B have comparable CC_50_ values of 5.39 and 4.31 μg/mL, respectively. However, the selectivity indices of amphotericin B (453 and 684) were much higher than anemonin (22 and 14) against *L. aethiopica* and *L. donovani*, respectively (Table 2), indicating that amphotericin B exhibits more selective toxicity to the parasites than anemonin.

As shown in Table 3, the in vitro antischistosomal assay showed that NTS were more susceptible to anemonin than adult *S. mansoni*, revealing high activity at 10 µM after 72 h of incubation.

Results of the present study suggest that anemonin might be responsible for the antileishmanial activity of the fresh leaves of *R. multifidus*. Several studies reported that anemonin possesses anti-inflammatory activity by different mechanisms of action [31,32,33,34,35,36,37]. There are also reports that anemonin exhibits antibacterial [38,39], wound healing [40], antioxidant [41] and neuroprotective [35] activities. Saidi et al. [40] reported that anemonin has potent wound healing activity making it a promising candidate as a therapeutic agent in tissue repairing processes.

Leishmaniasis is still one of the endemic and highly prevalent diseases in Ethiopia [42]. Most people in Ethiopia use traditional medicine for the treatment of cutaneous leishmaniasis and have no knowledge of modern medical treatment [2]. Even communities who have knowledge about modern medicine may not have access to healthcare service. This, coupled with the high costs of diagnosis and treatment, further enhances the spread of the disease and reinforce poverty [5]. In Ethiopia, people living in cutaneous leishmaniasis endemic areas are exposed to *L. aethiopica* infection, which leaves permanent scar causing disfigurement that results in social stigmatization [43,44,45]. Most cutaneous leishmaniasis patients seek folk medicine and the majority have the attitude that treatment from traditional healers is effective [46]. In different parts of Ethiopia, fresh leaves of *R. multifidus* are used for the treatment of cutaneous leishmaniasis (without leaving a scar) [17,18] and various types of skin diseases [13,47,48,49,50]. The present work confirms that *R. multifidus* extracts have the capacity to control localized cutaneous leishmaniasis caused by *L. aethiopica* and visceral leishmaniasis induced by *L. donovani*. Furthermore, inhibition of proliferation of the leishmania parasites by anemonin justifies the traditional use of the leaves of *R. multifidus* for the treatment of cutaneous leishmaniasis.

Although the prevalence of schistosomiasis in Ethiopia may have decreased over time due to the strategic use of anthelmintics [51], the disruption of the health system due to COVID-19 may have increased the prevalence. The WHO recommended the postponement of all mass drug administration (MDA) programs, community-based surveys and active case finding activities in April 2020. Schistosomiasis and visceral leishmaniasis were among the diseases that will be most affected by the disruption of the programs against NTDs [52]. Due to the potential rise in cases of schistosomiasis caused by disruption to control programs, the antischistosomal activity of anemonin was also investigated. Although the antischistosomal activity of anemonin may have been low compared to the antileishmanial activity, it shows the potential of the compound to have various antiparasitic activities.

## 3. Materials and Methods

### 3.1. Plant Material

Fresh leaves of R. multifidus were collected from Dorze village of Chencha woreda, Gamo zone (520 km southwest of Addis Ababa, Ethiopia), located in the Rift Valley above the west shore of Lake Abaya at 6°11′36″ N and 37°34′13″ E. The plant material was authenticated by Ato Melaku Wondafrash, National Herbarium, Department of Biology, College of Natural and Computational Sciences, Addis Ababa University (AAU), where a voucher specimen was deposited (collection number BS-001) for future reference.

### 3.2. Chemicals and Reagents

The chemicals and reagents used for the experiment include methanol (Cheshire, UK), Amphotericin B (Laborchemikalien GmbH, Germany), Giemsa (ESJAY Chemicals, Maharashtra, India), resazurin sodium salt, dimethyl sulfoxide (DMSO), triton X-114, potato starch powder (Sigma-Aldrich Laborchemikalien GmbH, Germany), phosphate buffer saline (PBS) (Gibco, Waltham, MA, USA), Roswell Park Memorial Institute-1640 (RPMI-1640) (Sigma-Aldrich, Gillingham, UK), minimum essential medium (MEM) (Sigma-Aldrich Co., St. Louis, MO, USA), heat inactivated new born calf serum (HINBCS) (Sigma-Aldrich Co., St. Louis, MO, USA) and penicillin-streptomycin solution (Sigma-Aldrich Co., St. Louis, MO, USA).

### 3.3. Experimental Animals and Parasites

Swiss albino mice were obtained from the animal house of the Department of Pharmacology (DoP), School of Pharmacy (SoP), College of Health Sciences (CHS), AAU. Mice of either sex weighing 22–30 g and age 5–6 weeks were employed. The animals were held in stainless steel cages at room temperature (20–22 °C) and a 12 h light/12 h dark cycle. They were provided with water and food pellets ad libitum in the animal house of the DoP, SoP, CHS, AAU. All the experiments were conducted in accordance with the internationally accepted laboratory animal use and care guideline [53] and were approved by the Institutional Review Board of the SoP, AAU (Approval code: ERB/SOP241b/13/2021). Clinical isolates of *L. aethiopica* (306/17) and *L. donovani* (139/19) were obtained from Leishmaniasis Research and Diagnostic Laboratory (LRDL), at the Department of Microbiology, Immunology and Parasitology, CHS, School of Medicine (SoM), AAU. The life cycle of *Schistosoma mansoni* is maintained at the Swiss Tropical and Public Health Institute (Swiss TPH).

### 3.4. Extraction and Isolation

Fresh leaves of *R. multifidus* were extracted by maceration using 80% methanol to obtain RM-M, and the hydro-distilled extract (RM-H) was prepared by hydro-distillation using Clevenger apparatus. Preparative TLC was used to isolate the major compound from the hydro-distilled extract. We obtained about 4 g of anemonin from 1 kg of fresh plant material. Extraction of anemonin has been reported previously by another method [54]. The characterization of the compound was achieved by means of spectroscopic techniques as described previously [26].

### 3.5. Antileishmanial Assay

#### 3.5.1. Leishmania Culture

Clinically isolated *L. aethiopica* and *L. donovani* parasites were cultured in Lock’s treated Novy-MacNeal-Nicolle (NNN) medium containing antibiotic solution (penicillin 100 IU/mL and streptomycin 100 µg/mL) [55]. The logarithmic stage parasites were transferred from NNN media into tissue culture flasks containing complete RPMI-1640 medium (RPMI-1640 medium supplemented with 10% heat inactivated foetal calf serum (HIFCS) and 100 IU penicillin/mL and 100 μg/mL streptomycin solution) at 22 °C for *L. aethiopica* and 26 °C for *L. donovani* [56,57]. The culture was monitored every day for two weeks to ensure healthy growth of parasites [58]. The logarithmic stage of the parasites was used for antipromastigote assay, while the stationary phase (metacyclic phase) was used for macrophage infection (antiamastigote assay) [55].

#### 3.5.2. Antipromastigotes Assay

To 96-well plates filled with 100 µL of complete RPMI-1640 medium, 100 µL of test substance (300 μg/mL) dissolved in 3% DMSO was added on the first well. Then, 100 µL was taken into subsequent wells then the last 100 µL was discarded, to achieve three-fold serial dilution. A total of 12 dilutions ranging from 100 µg/mL up to 0.000565 µg/mL were made to establish a full dose titration and determine IC_50_ values. All dilutions were done carefully by avoiding bubble formation. DMSO <1% was used as a negative control (this concentration was used to prevent its negative outcome in cell viability), and amphotericin B was used as a positive control (same serial dilution as the test substances) [59]. Then, 100 µL of suspension of logarithmic stage of the parasites (1 × 10^6^ promastigotes/mL of *L. aethiopica* or *L. donovani*) were added to each well and contents of the plates were incubated at 22 °C for *L. aethiopica* and 26 °C for *L*. *donovani* [58]. After 68 h of incubation 2 μL of fluorochrome resazurin solution (12.5 mg AlamarBlue dissolved in 100 mL of PBS) were added and incubated accordingly for 4 h for a total of 72 h [60]. The fluorescence intensity was then measured by microplate fluorometer and luminometer at excitation wavelength of 530 nm and emission wavelength of 590 nm [60]. The background fluorescence intensity of the complete media, samples and reference drug was measured and subtracted for the corresponding wells.

#### 3.5.3. Intra-Peritoneal Macrophage Collection and Culture

Macrophages were collected from mice first by disinfecting the skin around the peritoneum with 70% ethanol and a mouse was injected with 1 mL of freshly prepared 2% starch intraperitoneally [61]. After four days, the mice were sacrificed by spinal dislocation and the skin underlying the peritoneal cavity was shaved aseptically to expose the intact peritoneum. Then 10 mL of sterile ice-cold phosphate buffered saline (PBS) with 3% HINBCS were injected into the peritoneal cavity and the peritoneal wall was massaged carefully to dislodge the attached macrophages [62]. Macrophages were then collected by drawing 6–8 mL of exudates which were transferred into sterile 15 mL test tubes and centrifuged at 3500 rpm for 10 min at 4 °C. A portion of the resulting pellet was cultured in 96-well plates containing 1 × 10^4^ macrophage suspended in complete RPMI-1640 medium and kept for macrophage cytotoxicity assay [63].

#### 3.5.4. Antiamastigotes Assay

A portion of macrophages were suspended in complete MEM media containing 10% HINBCS, 2 mM L-glutamine, 100 IU penicillin and 100 μg streptomycin/mL. The host cells (macrophage) were counted and adjusted accordingly using a haemocytometer to 1 × 10^6^ cells per mL in complete MEM media. Then 300 μL (3 × 10^5^ macrophages) were seeded in every well of an 8-well plate containing removable microscopic slides. The cultured macrophage cells were allowed for adherence for at least 12 h at 37 °C in 5% CO_2_. Non-adherent cells were washed twice with pre-warmed complete MEM media and incubated overnight in fresh media [64]. Following overnight incubation, adherent cells were infected separately with late stationary stage *L. aethiopica* and *L. donovani* promastigotes with a parasite-to-cell ratio of 10:1 and incubated further for 12 h. After removal of non-internalized promastigotes by extensive washing with complete MEM media, cultures were left to rest for about 4 h [65]. Amphotericin B was used as a reference drug to check sensitivity of the parasites. Samples were serially diluted in 8-well plates as indicated in the antipromastigote assay. The prepared macrophage culture was incubated with or without 100 μL of test substance for three days at 31 °C (*L. aethiopica*) and 37 °C (*L. donovani*), 5% CO_2_ and 95% relative humidity. Following 72 h of incubation, the media was discarded, the slides were washed with PBS (prewarmed at 37 °C) and methanol was used for fixation for 15 min; then the slides were stained with 10% Giemsa for 15 min and washed with distilled water. The slides were then observed under microscope with oil immersion (100× objective). The number of amastigotes was determined by counting amastigotes in at least 50 macrophages in duplicate cultures [65]. Antiamastigote activity was determined by assessing the infection rate and parasitic load in both treated and untreated groups. Infection was considered adequate if more than 70% of the macrophages present in the negative control were infected. The total actual parasite burdens were calculated using the infection index shown below [66].

$$\%\mathrm{Infection}\;\mathrm{index}=\frac{\mathrm{No}\;\mathrm{of}\;\mathrm{infected}\;\mathrm{macrophage}}{\mathrm{Total}\;\mathrm{macrophage}\;\mathrm{counted}}\times \frac{\mathrm{No}\;\mathrm{of}\;\mathrm{amastigotes}\;\mathrm{in}\;50\;\mathrm{infected}\;\mathrm{macrophages}}{\mathrm{Total}\;\mathrm{macrophage}\;\mathrm{counted}}$$

The first half of the equation gives the percentage of infected macrophages and the second gives average parasite load found in infected macrophages [67]. The IC_50_ is inhibitory concentration of the test substance that reduces 50% amastigotes density [68].

#### 3.5.5. Macrophage Cytotoxicity Assay

Macrophage cells were cultured in complete RPMI-1640 media, humidified 5% CO_2_ and incubated at 37 °C for 24 h [62]. In 96-well plates containing a pre-cultured 100 µL suspensions of 1 × 10^4^ macrophage, the medium was replaced by 100 µL of serially diluted test substances. A total of 12 dilutions (1000 µg/mL–0.00565 µg/mL) dissolved in 1% DMSO were made [64]. Then contents of the plates were incubated at 37 °C in 5% CO_2_. After 48 h of incubation, 5 μL (1/20, *v*/*v*) fluorochrome resazurin solution was added into each well and the fluorescence intensity was measured after 3 h using microplate fluorometer and luminometer at excitation wavelength of 530 nm and emission wavelength of 590 nm [69]. The concentration which kills 50% of the cells for each test substance (CC_50_) was calculated [68].

#### 3.5.6. Selectivity Index

Selectivity index (SI) was determined using CC_50_ of the normal macrophage and the IC_50_ of amastigotes. The selectivity of the test substance in killing the parasites as opposed to mice macrophage cells was assessed by the following formula [63]:

$$\mathrm{Selectivity}\;\mathrm{index}\;\mathrm{(SI)}=\frac{{\mathrm{CC}}_{50}\;\mathrm{macrophage}}{{\mathrm{IC}}_{50}\;\mathrm{amastigote}}$$

### 3.6. Hemolysis Assay

Hemolytic activity was determined by using red blood cells (RBCs) prepared from freshly collected O^+^ human blood (2 mL) added to 48 mL of PBS and centrifuged at 3500 rpm for 10 min at 4 °C. The supernatant was washed off (3×) with PBS resulting in the formation of approximately 1 mL of RBC pellets [70]. The resulting pellet was then re-suspended in 49 mL of PBS to make 2% blood suspension and the concentration adjusted to 1.9 × 10^9^ RBC/mL. Then, 200 µL of the blood suspension was pipetted into Eppendorf tubes containing each test substance at concentrations of 3.7, 11.11, 33.33, 100, 300, and 900 μg/mL to give a final volume of 1500 µL [71]. The suspension containing 2.5 × 10^8^ RBC/mL was carefully mixed and incubated at 37 °C for 2 h. The membrane destabilizing activity of each test substance was determined in terms of its capability to rupture the cell membranes of RBC letting the release of hemoglobin into the solution. The mixture was centrifuged at 3500 rpm for 10 min resulting in intact and ruptured RBC to pellet liberating the hemoglobin in the supernatant solution, and 75 µL from the supernatant of each tube was collected in 96-well plates; absorbance was measured at 540 nm using a Lambda 9 spectrophotometer (Perkin Elmer, UK) [72]. Triton X-114 (5 µL/mL) was used as a positive control, and was prepared by adding 50 µL of blood to 100 µL Triton X-114 and incubated at 37 °C for 30 min [73]. RBC suspension with 1% DMSO was used as a negative control. Hemolytic effects were expressed as percentage of the absorbance of the positive control (100%) and the 50% lytic concentrations (LC_50_) [72,74,75].

### 3.7. Antischistosomal Assays

In vitro studies were carried out in accordance with Swiss national and cantonal regulations on animal welfare under the permission number 2070. The drug sensitivity assays with *Schistosoma mansoni* (adult and newly transformed schistosomules (NTS)) were carried out as described recently [76]. Briefly, to obtain NTS, cercariae were collected from infected *Biomphalaria glabrata* snails and were mechanically transformed. Adult *S. mansoni* worms were collected by dissecting the mesenteric veins of infected mice at day 49 post-infection. Approximately 30–40 NTS were incubated with the respective test drug in 250 μL of M199 medium (Gibco, Waltham, MA, USA) supplemented with 5% (*v*/*v*) foetal calf serum (FCS) (Bioconcept AG, Switzerland), 1% (*v*/*v*) penicillin/streptomycin solution (Sigma–Aldrich, Switzerland) and 1% (*v*/*v*) antibacterial/antifungal solution for up to 72 h at 37 °C and 5% CO_2_. The drug was tested at 10 and 1 μM in triplicate and repeated once. Three adult *S. mansoni* were incubated in a final volume of 2 mL RPMI 1640 supplemented with 5% (*v*/*v*) FCS and 1% (*v*/*v*) penicillin/streptomycin at 37 °C and 5% CO_2_ for 72 h at 10 μM. The experiment was conducted in duplicate.

Worms were judged via microscopic readout 72 h after incubation; they were scored according to phenotypic reference points such as motility, morphology and granularity (scores from 0 to 3).

### 3.8. Statistical Analysis

The antileishmanial, macrophage cytotoxicity and haemolysis assays were done in triplicate experiments with each test concentration in duplicate. The respective IC_50_, CC_50_ and LC_50_ values for each test substance were determined by non-linear regression analysis from sigmoidal dose–response curves using computer software GraphPad prism 8.0 and values were expressed as mean ± standard error of mean (SEM). Statistical significance was determined by one-way ANOVA followed by Tukey post hoc test to compare different parameters among the treatment and control groups. *P* < 0.05 was considered significant.

## 4. Conclusions

In conclusion, the results of the present study revealed that the fresh leaf extracts of *R. multifidus* display potent in vitro antileishmanial activity which justify the traditional use of the plant for the treatment of leishmaniasis. The finding also suggests that anemonin is likely responsible for the leishmanicidal activity of the plant. Anemonin also showed high antischistosomal activity against NTS and moderate activity against adult *S. mansoni*. To the best of our knowledge, this is the first study that reports the antileishmanial activity of *R. multifidus* and its major compound anemonin as well as the antischistosomal activity of anemonin. The promising activity of anemonin shown in this study warrants its potential as a lead compound for the development of safer, more potent and cost- effective alternative drugs for the treatment of leishmaniasis and schistosomiasis. Previously, we had shown that alkaline hydrolysis of anemonin results in the formation a dicarboxylate [26]. Therefore, one possibility is to use this hydrophilic anemonin derivative as a starting material and template for the synthesis of antileishmanial compounds. The formulation of anemonin in anti-inflammatory preparations gives indications as to how this lipophilic compound can be administered orally [77].

## Figures and Tables

**Table 1:** In vitro antileishmanial activity of the fresh leaf extracts of *Ranunculus multifidus* and anemonin against promastigote and amastigote forms *Leishmania aethiopica* and *Leishmania donovani*.

Test Substance	Antileishmanial Activity IC_50_ (μg/mL)			
	Promastigotes		Amastigotes	
	*Leishmania aethiopica*	*Leishmania donovani*	*Leishmania aethiopica*	*Leishmania donovani*
RM-M	14.92 ± 0.554 ^a1,b1,c1^	22.12 ± 0.564 ^a1,b1,c1^	17.487 ± 0.298 ^a1,b1,c1^	19.325 ± 0.24 ^a1,b1,c1^
RM-H	0.49 ± 0.004 ^b1,c1^	0.984 ± 0.028 ^b1,c1^	1.49 ± 0.004 ^b1,c1^	1.814 ± 0.028 ^b1,c1^
Anemonin	0.257 ± 0.007 (1.33) ^c1^	0.303 ± 0.304 (1.58) ^c1^	0.239 ± 0.014 (1.24) ^c1^	0.368 ± 0.024 (1.91) ^c1^
AMB	0.0157 ± 0.08 (0.017)	0.0067 ± 0.008 (0.0072)	0.0095 ± 0.004 (0.01)	0.0063 ± 0.011 (0.0068)

Data expressed as mean ± SEM; *n* = 3; a: compared to RM-H, b: compared to anemonin, c: compared to Amphotericin B; 1: *p* < 0.001; RM-M: 80% methanol extract of *R. multifidus*, RM-H: hydro-distilled extract of *R. multifidus*; AMB: amphotericin B. Values in parenthesis indicate concentration in nanomolar (nM).

**Table 2:** In vitro macrophage cytotoxicity, hemolytic property and selectivity indices of the fresh leaf extracts of *Ranunculus multifidus* and anemonin.

Test Substance	Cytotoxicity (μg/mL)		Selectivity Index	
	Macrophage (CC_50_)	Hemolysis (LC_50_)	*Leishmania aethiopica*	*Leishmania donovani*
RM-M	256.62 ± 0.211 ^a1,b1,c1^	>1000 ^a1,b1,c1^	14 ^a1,b1,c1^	13 ^a1,c1^
RM-H	4.98 ± 1.583 ^b1,c1^	25.68 ± 0.07 ^b1,c1^	3 ^b1,c1^	3 ^b1,c1^
Anemonin	5.39 ± 2.013 (28.00) ^c1^	91.00 ± 0.298 (473.95) ^c1^	22 ^c1^	14 ^c1^
AMB	4.31 ± 0.983 (4.66)	47.25 ± 0.54 (51.13)	453	684

Data expressed as mean ± SEM; *n* = 3; a: compared to RM-H, b: compared to anemonin, c: compared to Amphotericin B; 1: *p* < 0.001; RM-M: 80% methanol extract of *R. multifidus*, RM-H: hydro-distilled extract of *R. multifidus*; CC_50_: concentration causing 50% cytotoxicity, LC_50_: concentration causing 50% lysis; AMB: amphotericin B. Values in parenthesis indicate concentration in nanomolar (nM).

**Table 3:** In vitro antischistosomal activity of anemonin against adult *Schistosoma mansoni* and newly transformed schistosomula (NTS).

	NTS	NTS	Adult *Schistosoma mansoni*
Test Substance	% activity (72 h, 10 µM)	% activity (72 h, 1 µM)	% activity (72 h, 10 µM)
Anemonin	93.75 ± 2.1	41.38 ± 3.4	48.95 ± 2.0

## Data Availability

The authors declare that all data supporting the finding of this study are included in this article.

## Sample Availability

Samples of the compound are available from the authors.
